# Genome-Wide Association Mapping for Cold Tolerance in a Core Collection of Rice (*Oryza sativa* L.) Landraces by Using High-Density Single Nucleotide Polymorphism Markers From Specific-Locus Amplified Fragment Sequencing

**DOI:** 10.3389/fpls.2018.00875

**Published:** 2018-06-28

**Authors:** Jiayu Song, Jinqun Li, Jian Sun, Tao Hu, Aiting Wu, Sitong Liu, Wenjia Wang, Dianrong Ma, Minghui Zhao

**Affiliations:** ^1^Rice Research Institute, Shenyang Agricultural University, Shenyang, China; ^2^State Key Laboratory for Conservation and Utilization of Subtropical Agro-Bioresources, South China Agricultural University, Guangzhou, China; ^3^Department of Plant Breeding and Genetics, Max Planck Institute for Plant Breeding Research, Cologne, Germany

**Keywords:** rice (*Oryza sativa* L.) landraces, Ting’s rice core collection, cold tolerance, genome-wide association study, candidate gene, seedling stage

## Abstract

Understanding the genetic mechanism of cold tolerance in rice is important to mine elite genes from rice landraces and breed excellent cultivars for this trait. In this study, a genome-wide association study (GWAS) was performed using high-density single nucleotide polymorphisms (SNPs) obtained using specific-locus amplified fragment sequencing (SLAF-seq) technology from a core collection of landraces of rice. A total of 67,511 SNPs obtained from 116,643 SLAF tags were used for genotyping the 150 accessions of rice landraces in the Ting’s rice core collection. A compressed mixed liner model was used to perform GWAS by using the high-density SNPs for cold tolerance in rice landraces at the seedling stage. A total of 26 SNPs were found to be significantly (*P* < 1.48 × 10^-7^) associated with cold tolerance, which could explained phenotypic variations ranging from 26 to 33%. Among them, two quantitative trait loci (QTLs) were mapped closely to the previously cloned/mapped genes or QTLs for cold tolerance. A newly identified QTL for cold tolerance in rice was further characterized by sequencing, real time-polymerase chain reaction, and bioinformatics analyses. One candidate gene, i.e., *Os01g0620100*, showed different gene expression levels between the cold tolerant and sensitive landraces under cold stress. We found the difference of coding amino acid in *Os01g0620100* between cold tolerant and sensitive landraces caused by polymorphism within the coding domain sequence. In addition, the prediction of Os01g0620100 protein revealed a WD40 domain that was frequently found in cold tolerant landraces. Therefore, we speculated that *Os01g0620100* was highly important for the response to cold stress in rice. These results indicated that rice landraces are important sources for investigating rice cold tolerance, and the mapping results might provide important information to breed cold-tolerant rice cultivars by using marker-assisted selection.

## Introduction

Low temperature affects seed germination and male sterility of rice, and thus decreases yield (Koseki et al., 2010; Saito et al., 2010; Suh et al., 2010). Globally, over 15 million hm^2^ of rice is adversely affected by low temperature, especially in Japan, South Korea, North Korea, and Northeast China. With the rapid development of molecular biology, increasing number of studies have been focusing on the identification of quantitative trait loci (QTLs) responsible for cold tolerance in rice. Fujino et al. generated recombinant inbred lines (RILs) by crossing temperate *japonica* varieties Italica Livorno with Hayamasari and cloned a major gene, *qLTG3* on chromosome 3 (Fujino et al., 2004); this is the first cloned gene known to be associated with low-temperature germination in rice that encodes the unknown protein (Fujino and Matsuda, 2010; Fujino and Sekiguchi, 2011). The cold tolerance is a quantitative trait controlled by multiple genes in rice. Low temperature damage can occur in all developmental stages in rice. There were different genetic basis on cold tolerance at different developmental stages. Zhang et al. (2017) revealed that *CTB4a* was responsible for cold tolerance at the booting stage and cloned this gene, which encoded a conserved leucine-rich repeat receptor-like kinase. Saito et al. mapped a major QTL (*Ctb1*) on chromosome 4, which was found to be related to pollen fertility at the booting stage. The *Ctb1* locus only contained two candidate genes (F-box and protein serine/threonine kinase); molecular characterization showed that the F-box protein was the candidate gene of *Ctb1* and was highly expressed in young panicles (Saito et al., 2004).

At present, transplanting is still main way for rice sowing. The seedling establishment quality have a great influence on the growth and final yield of rice in the later period. At the same time, it is the high frequency suffer from low temperature damage at the rice seedling stage. Therefore, people began to focus on cold tolerance in rice seedling stage. Ma et al. (2015) found that *COLD1* gene confers cold tolerance in *japonica* rice at the seedling stage. They showed that *COLD1* is a GTPase-accelerating factor and regulates G-protein signaling in order to activate Ca^2+^ signaling under low temperature stress. Fang et al. knocked down of stress inducible *OsSRFP1* encoding an E3 ubiquitin ligase with transcriptional activation activity in rice, which showed expression content of *OsSRFP1* was inversely correlated with the cold tolerant levels, and confers abiotic stress tolerance through enhancing antioxidant protection in rice (Fang et al., 2015). Therefore, mining more valuable genes related to cold tolerance from a diverse rice germplasm resource is important since the complexity mechanism of cold tolerance.

The two subspecies of Asian cultivated rice (*Oryza sativa*) show differences in cold tolerance (Kovach et al., 2007): *japonica* cultivars grown in low-temperature regions generally possess stronger cold tolerance than *indica* cultivars (Tao and Song, 2007). Abundant germplasm resources for cold tolerance are available in the Asian cultivated rice, especially in rice landraces. As early as in 1920–1964, the famous Chinese rice researcher, Prof Ying Ting had collected more than 7128 accessions of rice landraces from all over China as well as from some main rice cultivation countries; this collection was named Ting’s collection. A rice core collection consisting of 150 accessions based on 48 agronomic traits from 2262 accessions of Ting’s collection has been constructed (Li et al., 2011). The abundant variation within the core collection provides an important reservoir of genetic diversity and potential sources of beneficial alleles associated with cold tolerance for rice breeding. A previous study indicated that the core collection of rice landraces showed abundant variation in cold tolerance (Wang et al., 2011). However, to our knowledge, no studies have used these rice collections to identify candidate genes for rice cold tolerance.

Recently, the whole genome sequencing technology is being increasingly used to accurately and rapidly detect numerous variants across the entire genome at the molecular level. SLAF-seq has been applied in genetic map construction, QTL mapping, and molecular breeding. However, no previous studies have used the SLAF-seq technology to develop genome-wide distributed markers for the analysis of polymorphisms, relationships, and population structures of a core collection of rice landraces. Genome-wide association study (GWAS) has emerged as a powerful approach for simultaneously screening genetic variations underlying complex phenotypes. Wang et al. found that genetic variation in *ZmVPP1* contributes to drought tolerance in maize seedlings (Mao et al., 2015; Wang X. et al., 2016). By using GWAS, Wang et al. also identified 67 QTLs for cold tolerance in rice at the seedling stage; these QTLs were located on 11 chromosomes of rice (Wang D. et al., 2016). In the present study, we perform GWAS for cold tolerance in the core collection by using high-density SNPs from SLAF-seq.

This study aimed to (1) use the SLAF-seq technology to develop genome-wide distributed markers from the rice core collection; (2) perform GWAS for rice cold tolerance to reveal the genetic basis for this complex trait; and (3) identify novel functional candidate genes associated with the mapped QTLs by using multiple molecular biological and bioinformatics approaches. These findings might provide an important basis for molecular breeding of rice cultivars having increased cold tolerance.

## Materials and Methods

### Plant Materials

The GWAS panel consisted of 150 accessions of landraces from Ting’s core collection. These landraces were mainly collected from 20 different provinces in China as well as from North Korea, Japan, Philippines, Brazil, Celebes, Java, Oceania, and Vietnam. These regions are distributed across the north latitude 55° to south latitude 10° and including regions with temperate, tropical, subtropical climate. Of the 150 landraces, 32 were classified as *japonica* rice (24 were typical *japonica* rice, and 8 were *japonica*-clined rice), and 118 were classified as *indica* rice (16 were *indica*-clined rice, and 102 were typical *indica* rice), according to Cheng’s index criterion (Zhang et al., 2011).

### Identification of Cold Tolerance Trait

The seeds were placed in a 50°C oven for 2 days to break dormancy. They were disinfected with 75% alcohol for about 20 min. Each accession had 48 seeds and grown for 20 days. The growth condition was 28°C and 2500 lux during daytime, and 24°C and 0 lux during nighttime. Recording the number of seedlings when the rice seedlings at three-leaf stage. Then seedlings were subjected to cold treatment which was divided into two stages: during the first stage, the plants were grown at 10°C for 7 days; during the second stage, they were allowed to recover at 28°C for 7 days. Three replicates were used for each treatment, and the mean of dead seedling percentage (DS) for the three replicates was calculated. The final result is the average of three biological repeats. The cold tolerance level were divided into five: level 1, 0% DS; level 3, 0.1 – 20.0% DS; level 5, 20.1 – 50% DS; level 7, 50.1 – 99.9% DS; and level 9, 100% DS (Yan et al., 1998).

### DNA and RNA Extraction

Genomic DNA was extracted from young leaves of the 150 accessions of landraces by using the CTAB method. The components of 100 mL CTAB buffer were 2 g CTAB, 1.4 M NaCl, 20 mM EDTA (pH 8.0), and 100 mM Tris–HCl (pH 8.0). DNA concentration and quality were estimated using a NanoDrop-2000 spectrophotometer (Thermo Fisher Scientific, Wilmington, DE, United States) and by electrophoresis on 0.8% agarose gels with a DNA marker.

Total RNA was extracted from young rice leaves by using RR420 (TaKaRa, Japan).

### SLAF Library Construction for High-Throughput Sequencing

For SNP genotyping, SNP-based polymerase chain reaction (PCR) amplification of derived cleaved amplified polymorphic sequence (Michaels and Amasino, 1998) markers was performed. Primers for the markers were designed using Primer Premier 5.0^1^ and dCAPS Finder 2.0,^2^ respectively. Each PCR contained 25 ng template DNA, 0.5 μM each of forward and reverse primers, 0.2 mM dNTPmix, 0.5 unit of Taq DNA polymerase, and 1 × PCR buffer (TaKaRa, Japan) in a total volume of 10.0 μL.

The GC content was used to design the markers by considering the enzyme digestion scheme, gel cutting ranges, and sequencing quantity.

We used the reference rice genome, IRGSP_build5. The SLAF library was constructed based on a pre-designed scheme established by referring to previous studies (Sun et al., 2013). The purified DNA tags with indices and adaptors (SLAFs) of 300–400 bp were used and diluted for pair-end sequencing on an Illumina High-seq 2500 sequencing platform according to the Illumina sample preparation guide (Illumina, Inc.; San Diego, CA, United States) at Beijing Biomarker Technologies Corporation.^3^ All polymorphic SLAF loci were genotyped according to the reference genome SNP loci. A group having more than two average depths for each sample was defined as a high-quality SLAF tag. The high-quality SLAF tags were used to construct a high-density genetic map.

#### Population Structure Analysis

Software ADMIXTURE (Alexander et al., 2009), which is based on the likelihood model embedded in software STRUCTURE, was applied to infer historical lineages that show clusters of similar genotypes. The membership of each genotype was run for the range of genetic clusters from a value of *K* = 1 to 10 by using the admixture model; for each *K*, the analysis was replicated five times. Each run was implemented with a burn-in period of 100,000 steps, followed by 100,000 Monte Carlo Markov Chain replicates. The software CLUSTER (Read and Vance, 2001) was used to perform principal component analysis to obtain the principal components of the 150 accessions of landraces. The phylogenetic tree for the 150 accessions of landraces was generated using the neighbor-joining method in MEGA5 software (Tamura et al., 2011).

### GWAS Analysis

Software TASSEL (Zhang et al., 2010) with compressed mixed linear model was applied for GWAS. Software ADMIXTURE (Alexander et al., 2009) was used to obtain the membership probability for each accession to determine the population structure. Software SPAGeDi (Ardy and Ekemans, 2002) was used to calculate the kinship matrix among all accessions. There are more than three significant SNPs with related to cold resistance in the ± 250Kb, which we define as a QTL.

### Candidate Gene Search

Previously cloned genes and mapped QTLs were screened in the flanking regions of the extremely significant loci (±200 kb) associated with cold tolerance from the QTARO database.^4^

The candidate genes were searched in the flanking regions of the extremely significant loci (±200 kb) associated with cold tolerance from the Rice QTL Map.^5^

### RT-PCR

To identify candidate genes and analyze their biological function and determine whether they are associated with cold tolerance, we investigated the expression levels of candidate genes by using RT-PCR. The cold tolerant and sensitive landraces were grown for 20 days at 28°C, and then were transferred to the environment at 28 and 4°C, respectively. We take in samples (0.1–0.2 g) rapidly in fixed temperature incubator under different of temperature at 20, 60 min, 12, and 18 h. The samples were stored at −80°C in darkness. Complementary DNA (cDNA) was synthesized using the primer from total RNA by using the First Strand cDNA Synthesis Kit from RR03A (TaKaRa). The RT-PCR primers (Supplementary Table S1) for amplification were designed using Primer 3.0 online.^6^ The landrace Mang (*japonica*, cold tolerant) and Danuo (indica, cold sensitive) were randomly chosen from the four most cold-tolerant landraces and the eight most cold-sensitive landraces, respectively. Two accessions were used for RT-PCR in this research. The reaction was performed on a 7500 Real-Time PCR system (Applied Biosystems, Carlsbad, CA, United States). The expression level of actin was used to standardize the RNA sample for each analysis. The RT-PCR assay was performed at least three times for each experimental unit and three biological repeats.

### Expression Patterns of Candidate Genes

The expression patterns of candidate genes at the seedling stage were determined by referring to the public database from TENOR: Database for comprehensive mRNA-Seq Experiments in Rice.^7^ The method involved obtaining genome-wide transcriptional activity data at single-base resolution and stress-responsive expression profiles for shoots and roots of rice. In all, 12 types of environmental stress (e.g., drought, cold, and flood), nutrient (phosphate) deficiencies and excesses, heavy metal (cadmium) toxicity stress, and plant hormone (abscisic acid and jasmonic acid) treatments were included.

### Protein Three-Dimensional Structure Analysis

Sequence analysis of proteins for the candidate genes was performed using software MEGA 5 (Tamura et al., 2011). The three-dimensional structure was determined using online software SMART.^8^ Alignments executed by the software were mostly based on the published researches. For the protein database, the “Genomic” mode in software SMART was used, which contained the data from completely sequenced genomes only. The Ensembl database was used for Metazoan genomes, and Swiss-Prot database was used for the other genomes. Mang, Danuo, Zjingu (*japonica*, cold tolerant), Bachongsui (indica, cold sensitive) were be used for sequencing of candidate genes.

## Results

### Phenotypic Variation

**FIGURE 1:**
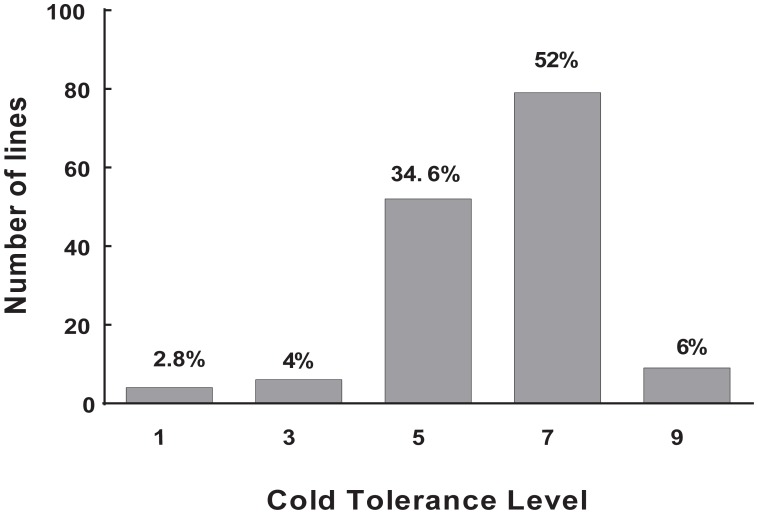
The distribution of cold tolerance for the 150 accessions of landrace in the Ting’s rice core collection. level 1–level 9: level 1, 0% DS; level 3, 0.1 – 20.0% DS; level 5, 20.1 – 50% DS; level 7, 50.1 – 99.9% DS; and level 9, 100% DS. The *Y*-axis represents number of accessions and *X*-axis represents cold tolerance level. The value on each bar represents the percentage of the total accessions of the core collection.

Cold tolerance was measured using plant DR at the rice seedling stage under cold stress, because this trait shows a relatively high reproducibility. Thus, temperature limitation was the predominant stress, compared to other possible environmental fluctuations or developmental nuances. Large variations in DS ranging from 100 to 0% were observed in repeated phenotypic assays. The core collection showed large variation for cold tolerance, which ensured the diversity of the target trait in the population. This result is consistent with that of our previous study (Zhang et al., 2011). The frequency of DS showed a normal distribution among the 150 accessions of landraces. The largest number was 79 accessions (52%) in 7L, whereas the least number was four accessions (2.8%) in 1L, six accessions (4%) in 3L, and nine accessions (9%) in 9L (**Figure 1**). These results showed that most landraces were sensitive to cold stress. Nonetheless, some landraces had high tolerance to cold stress, and these materials are an important source for breeding cultivars with cold tolerance. Finally, we obtain two accessions with extremely cold-tolerant and eight accessions with extremely cold-sensitive. Among them, the landrace Mang (*japonica*, cold tolerant) and Danuo (*indica*, cold sensitive) were used for RT-PCR and Mang, Danuo, Zjingu (*japonica*, cold tolerant), Bachongsui (*indica*, cold sensitive) were be used for candidate genes identification by sequencing.

### Analysis of SLAF-Seq Data

In total, we obtained 130,108,909 initial reads with the average read length of 160 bp after SLAF library construction and DNA high-throughput sequencing. The average percentage of Q20 base (a quality score of 20 indicating a 0.1% chance of an error and 99.9% confidence) ratio was 90.25%, and the average GC content was 46.5%.

**FIGURE 2:**
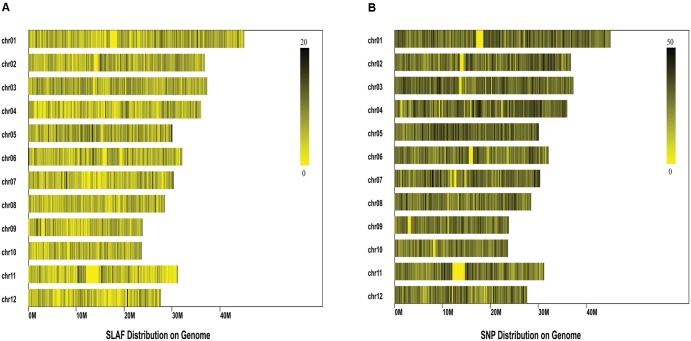
Distribution of the Specific-Locus Amplified Fragment **(A)** and SNPs **(B)** on the 12 rice chromosomes. Each row represents one chromosome and each vertical bar at the chromosome represents an interval of 100 K window. The ruler on the top right of each figure shows the density of SLAF/SNPs within each interval.

Comparison between the initial reads and reference genome (IRGSP_build5) revealed PE-Uniq reads (pair-end present, the percentage of two terminal sequences with a unique location in the genome) and SE-Uniq reads (single-end present, the percentage of single terminal sequence with a unique location in the genome) of 66.55% and 11.37%, respectively. The PE-Uniq reads were reliable and defined as SLAF tag loci. Finally, a total of 116,643 high-quality SLAFs were detected, with 24,889 polymorphic SLAF tags and polymorphism rate of 21.34%. The average sequence depth of each SLAF tag were 5.2. Statistical results showed that the SLAF tags were distributed unevenly on the 12 chromosomes of rice (**Figure 2A**). To sequence the inner region of SLAF tags, we detected 67,511 SNPs based on the SLAF tags (**Figure 2B**). The integrity ratio of these SNPs was 96.2% (which indicated high quality of these SNPs), and the heterozygous ratio was 10.86%.

### Population Structure Analysis

**FIGURE 3:**
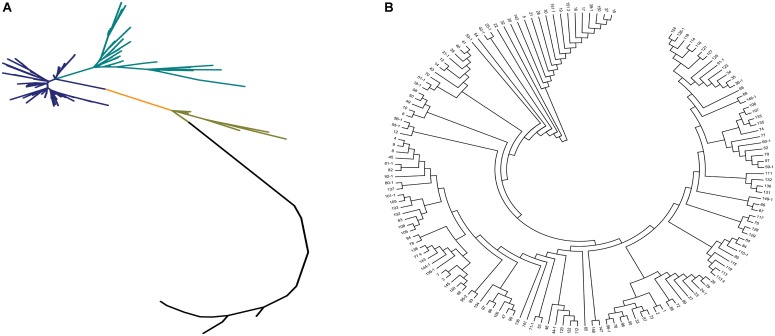
The phylogenetic tree of the 150 accessions of landraces in the Ting’s core collection. **(A)** The neighbor-joining trees for the entire population. Each branch represents an accession of landraces and two vertex distant represents the genetic relationship between the two landraces and each color represents a class of closely related species. **(B)** The cluster analysis of the 150 accessions of landraces.

Neighbor-joining method, STRUCTURE, and principal component analysis were used to analyze the population structure of and genetic relationships among the core collection. Based on the 67,511 SNPs, we constructed a phylogenetic tree by using the neighbor-joining method. The phylogenetic tree showed that the 150 accessions could be divided into 2–4 subgroups (**Figure 3A**). Two subgroups with the maximum distance were clustered in accordance with the differentiation of *indica* and *japonica* rice (**Figure 3B**).

**FIGURE 4:**
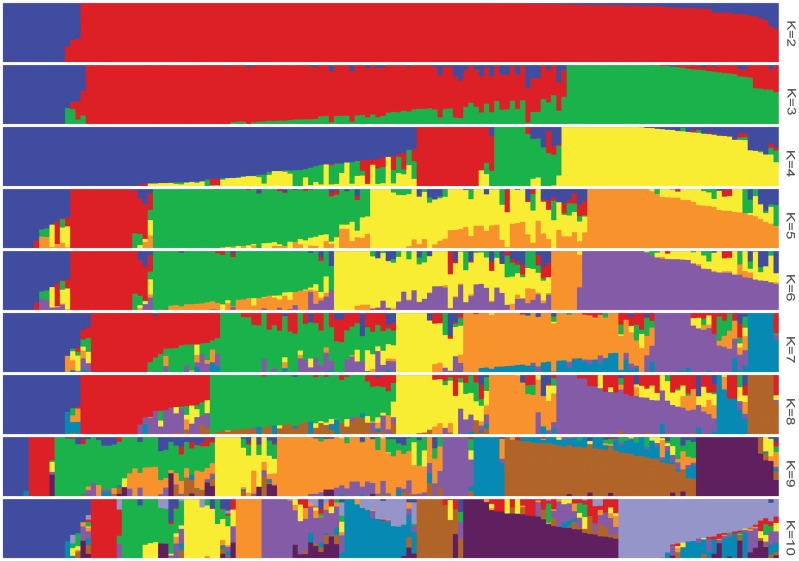
The population structure of the Ting’s rice core collection of landraces. The height of each bar represents the membership probability of one genotype assigned to different subgroups.

**FIGURE 5:**
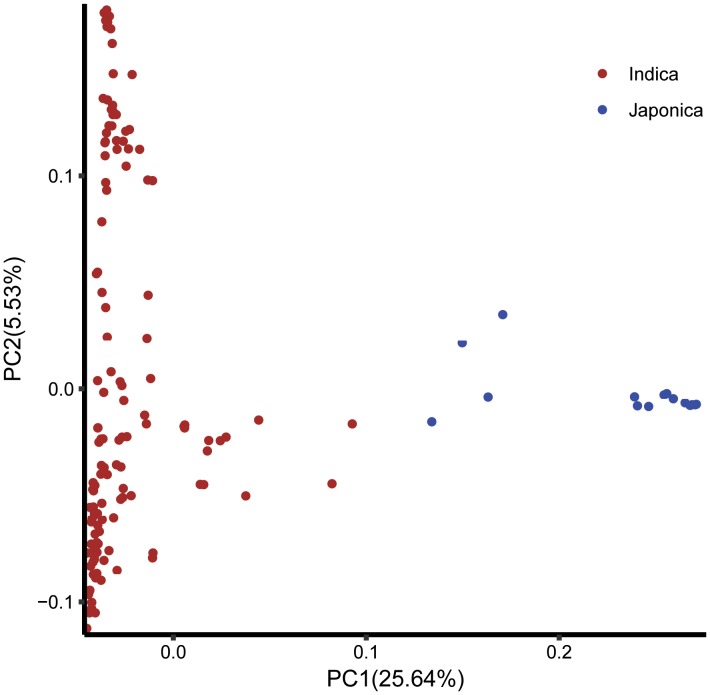
The principal component analysis based on the entire SNP set for the entire core collection. PC1 and PC2 refer to the first and second principal components, respectively. The numbers in parentheses refer to the proportion of variance explained by the corresponding axis.

The log likelihood revealed by STRUCTURE increased gradually from *K* = 1 to *K* = 10. The maximum *ad hoc* measure Δ*K* was observed for *K* = 2, which indicated that the entire population could be divided into two subgroups (**Figure 4**). Compared to the *indica*-*japonica* classification by Cheng’s index method (Zhang et al., 2011), the 15 *japonica* landraces were assigned to subgroup 1 and 135 *indica* landraces were assigned to subgroup 2 according to the membership probabilities of ≥0.7. The allele frequency was calculated using each marker for each landrace and used for principal component analysis. The result also revealed two distinct clusters for the entire population (**Figure 5**), which was related to their classification as *indica* and *japonica* types.

### GWAS for Cold Tolerance

**FIGURE 6:**
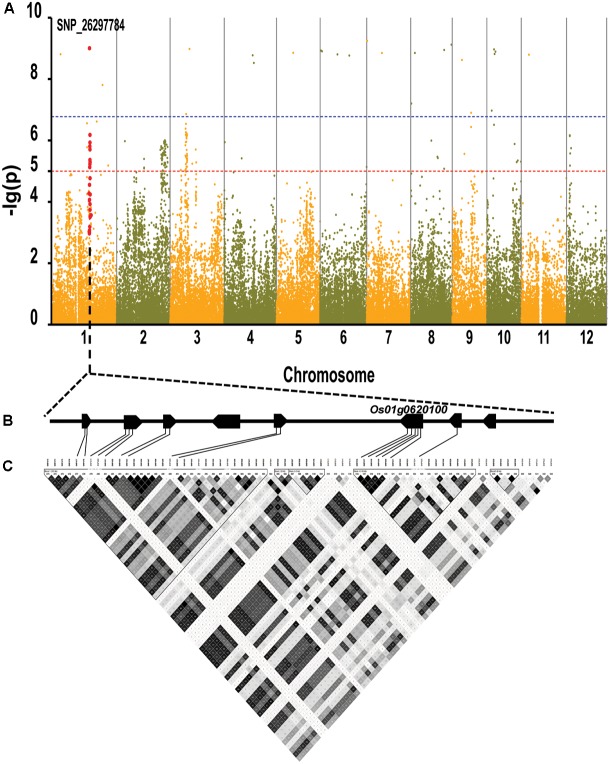
Genome-wide association mapping results for cold tolerance at seedling stage with the 150 accessions of landraces in the Ting’s rice core collection. **(A)** Manhattan plots of GWAS results with genetic association. The blue dashed line represents the Bonferroni significance threshold at *P* < 0.01 (with an empirical *P* < 1.48 × 10^-7^) and the red dashed line represents the significant threshold of *P* < 1.48 × 10^-5^. The red vertical lines mark the polymorphic sites identified by high-throughput sequencing. **(B)** Gene model of QTL. Filled black boxes represent eight candidate genes. **(C)** A representation of pairwise *r*^2^ values (a measure of LD) among all polymorphic sites in QTL, where the darkness of the color of each box corresponds to the *r*^2^ value according to the legend.

**FIGURE 7:**
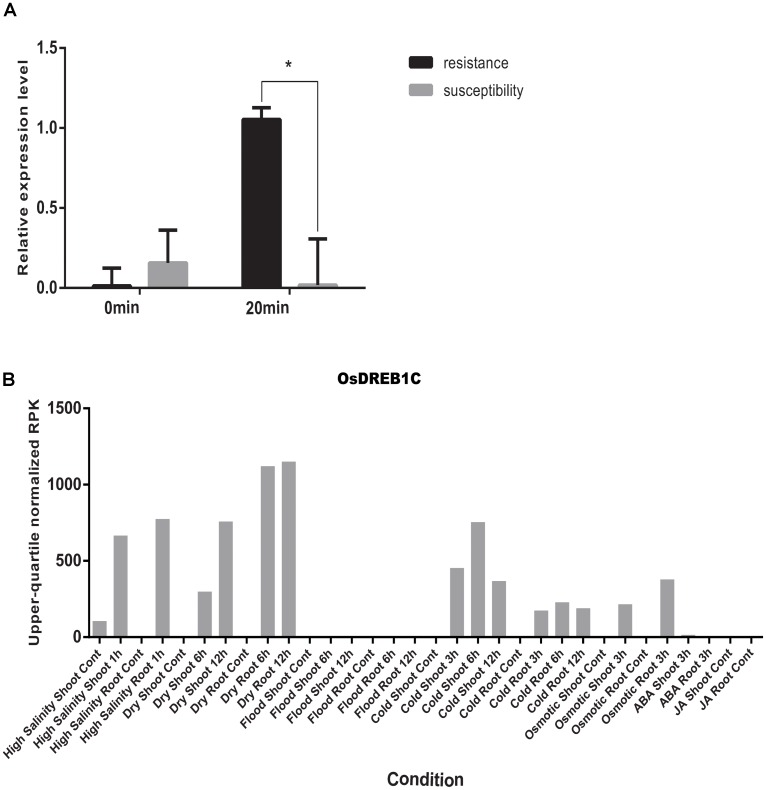
**(A)** The relative expression of *OsDREB1C* after cold stress in tolerant and sensitive landraces. Statistical significance was determined by a two-sided *t*-test: ^∗^*P* < 0.05. **(B)** Expression patterns of the *OsDREB1C* in rice with high salinity, high and low phosphate, high, low, and extremely low cadmium, drought, osmotic, cold, and flood and two plant hormone treatment conditions (ABA and Jasmonic Acid).

By using the compressed mixed liner model, we performed GWAS by using the high-density SNP set of 67,511 SNPs to identify the genetic loci underlying cold tolerance. An extremely significant threshold of *P* < 1.48 × 10^-5^ was used to determine that a total of 213 SNPs were associated with cold tolerance (data not shown). There are more than three significant SNPs in the ±250 Kb, which we define as a QTL. We mapped 22 QTLs on 12 chromosomes, of which three QTLs were co-localized with previous studies (*OsFAD2, OsMYB2*, and *OsCIPK03*) (Supplementary Table S2) (Xiang et al., 2007; An et al., 2012; Guan et al., 2012). Among of the three gene, the *OsMYB2* and *OsCIPK03* gene had been cloned and related to low temperature tolerance at rice seedling stage. In addition, the Bonferroni significant threshold at *p* value < 0.01 (*P* < 1.48 × 10^-7^) was used to determine that a total of 26 SNPs were significantly associated with cold tolerance (Supplementary Table S3). They were distributed across 10 chromosomes of rice except chromosomes 2 and 12 (**Figure 6A**). The explained percentage by QTL to the phenotypic variation ranged from 26 to 33%, which indicated that the QTL with the largest explained percentage (33%) was SNP_200936484 on chromosome 6. Within ±200 Kb of two SNPs (*P* < 1.48 × 10^-7^), *qCTB8* and *OsDREB1C* were detected, respectively. The *qCTB8* was found at 130 Kb downstream of SNP_2824184 (*P* = 1.41 × 10^-9^) at chromosome 8. The *qCTB8*, a quantitative trait locus for cold tolerance at the booting stage, was identified previously by using an over-expression approach in transgenic *Arabidopsis* based on the map-based cloning results by using RILs (Dubouzet et al., 2003). The *OsDREB1C*, one cloned gene, was found at 62 Kb downstream of SNP_1320300 (*P* = 1.25 × 10^-9^) at chromosome 6 (Kuroki et al., 2007). The *OsDREB1C* gene was a cold resistance gene at the seedling stage in rice and encoded transcription activators that function in drought-, high-salt- and cold-responsive gene expression (Dubouzet et al., 2003). We further analyzed the expression of *OsDREB1C* in one tolerant and one sensitive accessions at the seedling stage. The RT-PCR results showed that the expression of *OsDREB1C* (**Figure 7A**) was not significantly different between the cold tolerant and sensitive landraces at 28°C. However, after cold stress (at 10°C), its expression was significantly higher in the cold tolerant landraces than in the cold sensitive ones. The expression profiles from the database for comprehensive mRNA-Seq experiments in rice (Kawahara et al., 2016) indicated that the expression of *OsDREB1C* is higher in the shoot and root under cold as well as high salinity, high cadmium, drought, and flood stress (**Figure 7B**). Thus, our finding is consistent with those of previous studies (Liu et al., 2000; Tao et al., 2012).

**FIGURE 8:**
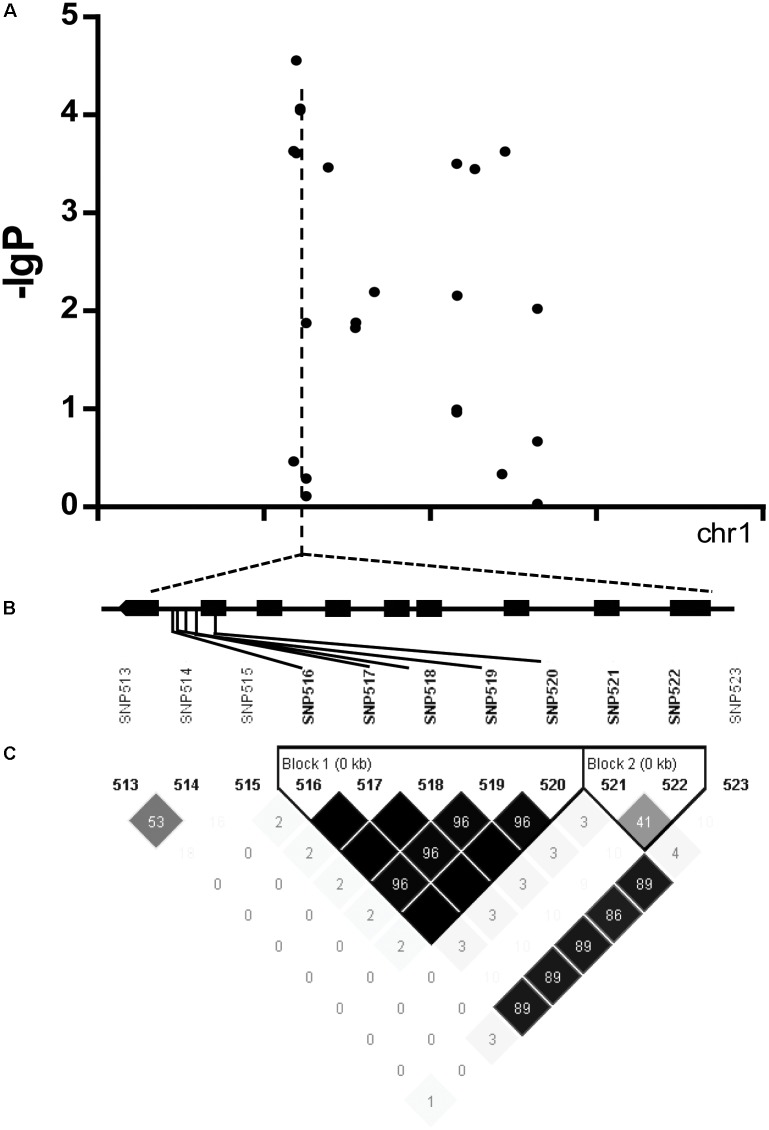
**(A)** Manhattan plot displaying the GWAS result of the content of trigonelline. **(B)** Gene model of *Os01g0620100*. Filled black boxes represent coding sequence. The gray vertical lines mark the polymorphic sites identified by high-throughput sequencing, and the star represents the proposed functional site. **(C)** A representation of pairwise *r*^2^ values (a measure of LD) among all polymorphic sites in *Os01g0620100*, where the darkness of the color of each box corresponds to the *r*^2^ value according to the legend.

We found there were three peaks corresponding to SNP_26297784, SNP_322940, and SNP_27990046 which were observed in the Manhattan plot, indicating very strong signals of trait-marker association at these regions, especially for SNP_2629778 on chromosome 1 (QTL2) which explained variation to phenotypic variation of 27%. The haplotype block analysis showed a strong linkage disequilibrium in this candidate QTL region (*R*^2^ > 0.95) (**Figure 8**). We will focus on the SNP_2629778 for further analysis.

### Candidate Gene Prediction and Validation

**FIGURE 9:**
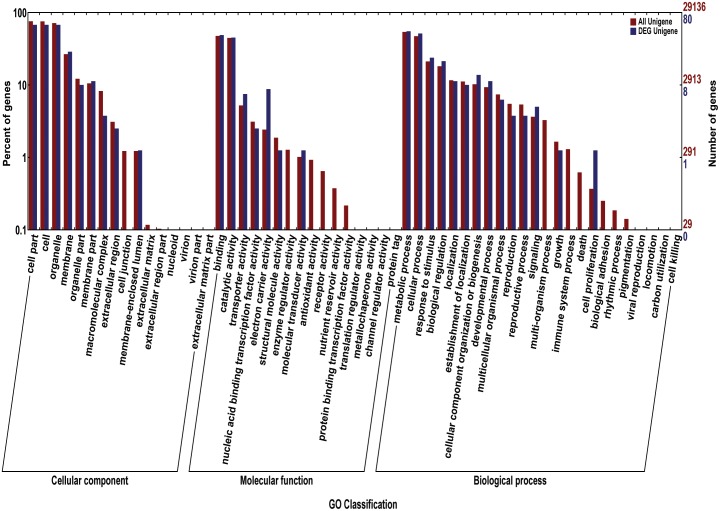
The gene ontology (GO) for the candidate genes. The red bars reprent percentage of genes (left *Y*-axis) for different classifications of ontology (cellular component, Molecular Function, and biological process) (*X*-axis); The blue bars reprent the number of genes (left *Y*-axis) for different classifications of ontology.

**FIGURE 10:**
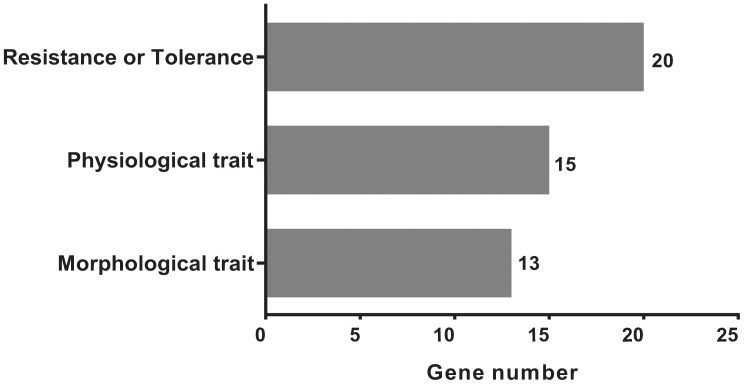
The classification and distributions of the candidate genes under the mapped positions for cloned genes and QTLs by searching the flanking region of 26 genetic variants significantly extremely loci (±200 Kb).

**FIGURE 11:**
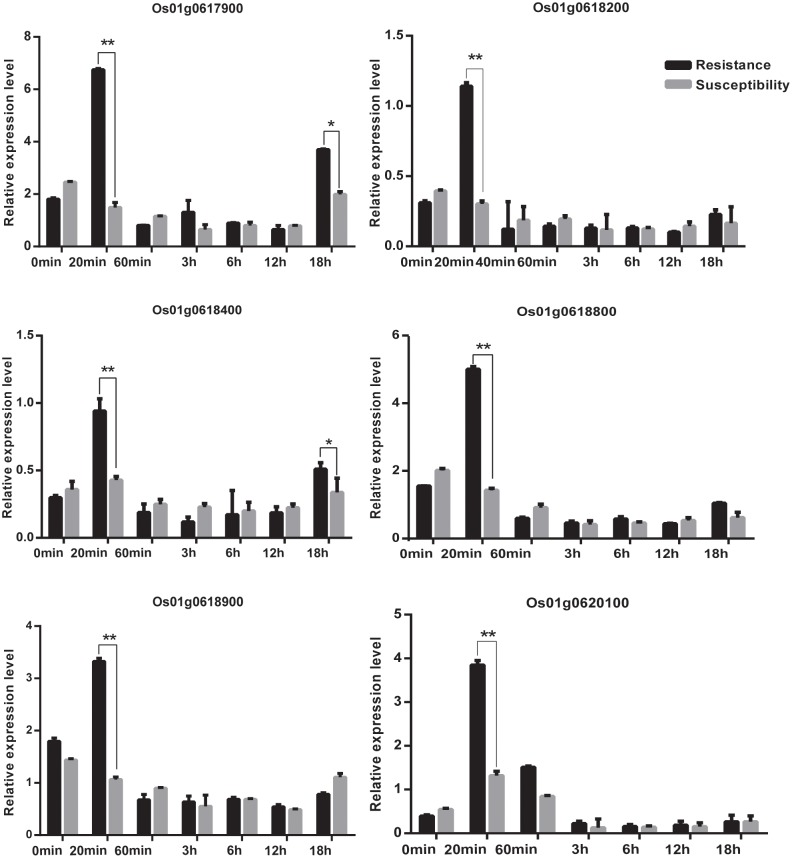
The relative expression of genes at 0 min, 20 min, 60 min,12 h,18 h at 4°C. Statistical significance (marked with ^∗∗^) was determined by a two-sided *t*-test at *P* < 0.01.

We acquired a total of 1023 candidate genes underlying all mapped SNPs by searching the flanking region of 26 extremely significant SNP loci (±200 Kb; data not shown). Gene ontology classified the candidate genes into three parts, i.e., cellular component, molecular function, and biological process, which also suggested proteins corresponding to cold resistance, protein containing kinase-like domain, and other known and unknown proteins (**Figure 9**). Among them, 25 genes had been cloned, and 23 QTLs had been mapped by previous studies. Further, 19 of 48 genes were found to play a role in resistance or tolerance (**Figure 10**). Since a significant peak was noted in the region of SNP_26297784 on chromosome 1, we further screened candidate genes within ±200 Kb of the mapped SNP and obtained 63 candidate genes (**Figure 6B**). We validated most of these candidate genes by using RT-PCR at 4°C at 0, 20, 60 min, 12, and 18 h after cold stress. Finaly, six of them, i.e. *Os01g0617900, Os01g0618200, Os01g0618400, Os01g0618800, Os01g0618900*, and *Os01g0620100*, changed significantly after cold treatment (**Figure 11** and Supplementary Table S1). Further, these six candidate genes have not yet been localized and cloned in rice.

**FIGURE 12:**
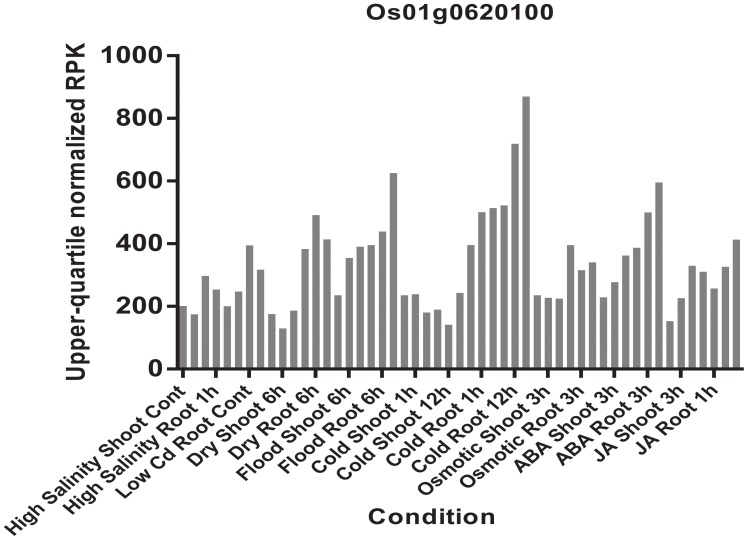
Expression patterns of the *Os01g0620100* in rice under high salinity, high and low phosphate, high, low, and extremely low cadmium; drought, osmotic, cold, and flood and two plant hormone treatment conditions (ABA and Jasmonic Acid). Each color represents different abiotic stress conditions in shoot and root of rice.

The haplotype blocks analysis of the QTL2 showed five SNPs with high linkage disequilibrium located at *Os01g0620100* (**Figure 8**). The gene annotation of *Os01g0620100* showed that it was the homolog of a gene in *Arabidopsis* associated with disease resistance, which has been cloned. In addition, the Os01g0620100 protein sequence in rice was highly similar to those from other plants such as short stalked grass (*Bradi2g43780.1)*, maize (*GRMZM2G057853*), sorghum (*Sb03g028180.1*), and *Arabidopsis thaliana* (*Seh1*), indicating that this gene was conserved in these species (Li et al., 2014). In addition, expression profile analysis in public databases from comprehensive mRNA-Seq experiments in rice showed that *Os01g0620100* was highly expressed under the cold stress (**Figure 12**). Interestingly, the expression level of *Os01g0620100* also increased under other environmental stresses such as high salinity, osmotic, drought, and flood. We speculated that *Os01g0620100* might be involved in the regulation mechanism of several stresses in addition to cold stress.

The six candidate genes were sequenced; *Os01g0620100* was found to contain an obvious mutation region (from 444 to 620 bp), and the other seven candidate genes had several single-base mutation sites in the cold tolerant landraces. We found that the coding sequence of *Os01g0620100* had one more domain between 444 and 620 bp region in the cold tolerant landraces, but not in the cold sensitive ones.

**FIGURE 13:**
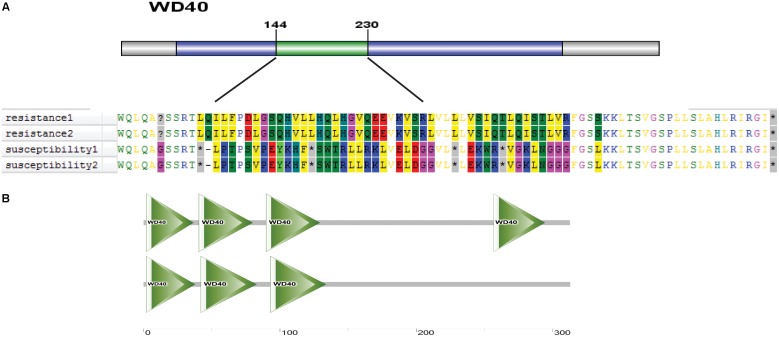
**(A)** A distinct amino acid sequence on the WD40 protein in cold tolerant and sensitive landraces. **(B)** Predicted three-dimensional structure of protein of Os01g0620100 genes.

Protein structure is closely related to plant phenotype. Therefore, we further analyzed the protein domain of *Os01g0620100* and predicted its three-dimensional structure. The difference in the coding sequence led to a different amino acid sequence in the region between 144 and 230 aa (**Figure 13A**). The Os01g0620100 protein included a WD40 domain, which was found in many eukaryotic proteins associated with adaptor/regulatory modules in signal transmission, pre-mRNA processing, and cytoskeleton assembly functions (**Figure 13B**). In addition, the three-dimensional structure of the protein was predicted using SMART online software, which also showed the WD40 protein domain that was frequently noted in cold tolerant landraces, but not common in the cold sensitive ones (**Figure 13B**). Thus, we speculated that *Os01g0620100* could be involved in the regulation of cold stress at the rice seedling stage.

## Discussion

Asian cultivated rice (*O. sativa*) was domesticated from its wild relatives *O. nivara* and O. *rufipogon* (Kovach et al., 2007; Sang and Ge, 2007). During human selection, cultivated rice has undergone significant changes in agricultural traits, for example, grain yield and environmental stress-related genes such as *SH4* (Huang et al., 2012) and *PROG1* (Xu et al., 2012). The signatures of domestication in cultivated rice have been identified using genetic mapping for QTLs and GWAS as well as population genetics. Because of domestication and artificial selection of rice, genetic diversity has been remarkably reduced in many cases, and favorable alleles or genes might have been lost in the modern cultivars. Rice landraces are the intermediate form between modern cultivars and their ancestral species. Because of the less impact by artificial selection, the intermediate forms contain abundant genetic diversity and useful elite genes for modern cultivars. Moreover, transfer of beneficial genes from the intermediate forms to modern cultivars is considerably easier than from the ancestral wild species.

Therefore, the identification and utilization of valuable genetic resources in landraces can be highly valuable for the genetic improvement of modern rice cultivars, for example, breeding cultivars for cold tolerance.

The development of molecular markers has remarkably promoted the progress in genotyping technologies (Vos et al., 1995). High-throughput sequencing technologies offer new methods for sequence-based SNP genotyping, which is more effective for genetic mapping and genome analysis (Huang et al., 2009). SLAF-seq combines high-throughput and reduced representation library sequencing, which is considered as an efficient and high-resolution strategy for large-scale genotyping (Sun et al., 2013). According to sequencing results, high-density SNP markers can be developed directly. Because of its high-throughput and cost–effective nature, SLAF-seq is an ideal method for genotyping by sequencing and hence has been applied in this study. This method allowed us to obtain a total of 67,511 high-quality SNPs that were used for further analysis.

Population structure of the core collection has been investigated by previous studies by using 274 simple sequence repeat (SSR) markers (Zhang et al., 2011). In our study, the phylogenetic tree showed that the entire population was divided into 2–4 subgroups (**Figure 3A**), unlike the two subgroups reported previously for these landraces. This difference can be attributed to the use of different methods (SSR genotyping vs. genotyping by sequencing via SLAF-seq). The high-density SNP markers from SLAF-seq had considerably higher coverage than SSR makers and could allow efficient detection of missing sites of SSRs. The influence of population structure might increase the false-positive rate (Atwell et al., 2010). In order to restrict the number of false-positives resulting from genetic structure, we used a compressed mixed linear model to reduce the impact of genetic relationships and population structure (Zhang et al., 2005; Yu et al., 2006). The conservative significant threshold, i.e., Bonferroni correction, was used as the significant threshold in this study to further reduce the false-positive rate.

Cold tolerance is a complex quantitative trait, which is affected by both stress time and severity exerted on plant growth and development. In addition to a transgenic approach, the selection and accumulation of tolerant or superior alleles of key genes functioning in stress tolerance are effective for the genetic improvement of rice. We found abundant variation in the core collection of rice landraces, among which a majority were cold-sensitive type, whereas some landraces were extremely cold tolerant. We identified 26 SNPs that were significantly associated with cold tolerance. By searching the flanking region (±200 Kb) of the mapped SNPs, we obtained 25 candidate genes that had been cloned, and 23 genes that had been mapped (**Figure 10**). The function of these genes included responsive gene expression under cold, drought, high salt, submergence, lodging, and blast stresses. The results showed that the significantly associated variant loci might participate in the cold tolerance mechanism of rice. Among them, the mapped SNP_2824184 (*P* = 1.41 × 10^-9^) on chromosome 8 was located 130 kb upstream of the previously mapped QTL *qCTB8* (Dubouzet et al., 2003) for cold tolerance in rice. The mapped SNP_1320300 (*P* = 1.25 × 10^-9^) on chromosome 6 was located 62 kb upstream of the previously cloned gene *OsDREB1C* for cold tolerance in *Arabidopsis.* Zhang et al. (2011) showed that linkage disequilibrium decay with genetic distance was about 200–500 kb in the core collection. Therefore, we considered that the mapping results for SNP_2824184 and SNP_1320300 were consistent with those from previous studies. However, other SNPs significantly associated with cold tolerance were not reported by previous studies; these might be the new loci controlling cold tolerance in rice at the seedling stage.

Among the newly identified loci for rice cold tolerance, a significant peak was noted in the regions of SNP_26297784 on chromosome 1. The SNP was selected for further screening of candidate genes within ±200 Kb of the SNP. The RT-PCR results showed that the relative expression level of eight candidate genes, i.e., *Os01g0617900, Os01g0618200, Os01g0618400, Os01g0618800, Os01g0618900*, and *Os01g0620100*, changed significantly after cold treatment at 4°C (**Figure 11** and Supplementary Table S1). The six candidate genes were further sequenced, and *Os01g0620100* was found to contain an obvious mutation region (from 444 to 620 bp), and the other candidate genes had several single-base mutation sites in the cold tolerant and sensitive landraces. The mutations might cause unusual expression of the cold tolerant genes.

*R* genes play an important role in cold stress. At present, transcription factors related to calmodulin-binding transcription activator (CBF), v-myb avian myeloblastosis viral oncogene homolog (MYB), and inductor of CBF expression were involved in the regulation of the molecular mechanism of cold tolerance (Lei et al., 2014). CBF and MYB are associated with two different cold tolerance pathways, and the former is inhibited when the latter transcription factor is expressed. The expression of CBF is rapid and for a short time (15 min after cold treatment), whereas the expression of MYB increases 12 h after cold treatment. In this study, we found that the expression of *Os01g0620100* rapidly increased and reached the highest level in 20 min, and then decreased to a level and remained almost unchanged after 60 min after cold treatment. We speculated that the *Os01g0620100* might be a CBF-like transcription factor to regulate the molecular mechanism of cold tolerance.

We further analyzed the sequence of *Os01g0620100* protein and predicted its three-dimensional structure. The protein structure of the gene was different in the cold tolerant and sensitive landraces. Thus, our results indicated that *Os01g0620100* could play an important role in the mechanism of cold tolerance in rice. A CRISPR/Case9 approach will need to be applied to re-edit the candidate gene and generate mutation lines to further characterize the function of the candidate rice cold tolerant genes.

## Author Contributions

MZ and DM designed the study. JL provides experimental materials. JyS, TH, WW, and AW analyzed the rice seedling phenotype. JyS and JS performed the necessary GWAS and phenotype analyses. JyS, TH, and SL performed the finding genes and qRT-PCR experiments. JyS, JL, JS, and TH analyzed the results and prepared the figures and tables. JyS and JL wrote the paper. All authors discussed the results and commented on the manuscript. All authors read and approved the final manuscript.

## Conflict of Interest Statement

The authors declare that the research was conducted in the absence of any commercial or financial relationships that could be construed as a potential conflict of interest.

## Footnotes

1 http://www.premierbiosoft.com/

2 http://helix.wustl.edu/dcaps/dcaps.html

3 http://www.biomarker.com.cn

4 http://qtaro.abr.affrc.go.jp/index.html

5 http://qtaro.abr.affrc.go.jp/cgi-bin/gbrowse/Oryza_sativa/?name=chr1

6 http://primer3.ut.ee/

7 http://tenor.dna.affrc.go.jp

8 http://smart.embl-heidelberg.de/
