# Supplementary material for: Genome-Wide Association Mapping for Cold Tolerance in a Core Collection of Rice (Oryza sativa L.) Landraces by Using High-Density Single Nucleotide Polymorphism Markers From Specific-Locus Amplified Fragment Sequencing
Source: Front Plant Sci. 2018 Jun 28;9:875. doi: 10.3389/fpls.2018.00875 (PMC6036282; doi:10.3389/fpls.2018.00875)
Supplement: Supplementary file 1 [file Table_1.doc]

Table S1. The primers used for quantitative real-time polymerase chain reaction

| **Gene** | **Forword Primer (5'-3')** | **Reverse Primer (5'-3')** |
| --- | --- | --- |
| actin | CCACACCCCTGCTATGTACG | CATCACCAGAGTCCAACACAA |
| Os01g0617900 | CCTCTGCTGCACCACTATCT | TCCCACTTGCTCTTGTCCTT |
| Os01g0618200 | CGAGGCTAGGTACATTTGCG | GTGAACCACCATGACCATCA |
| Os01g0618400 | AAAGCCGTCAAAGATGCTGG | TCCCAGTTCCTGTCTTTGCT |
| Os01g0618800 | CCTTGGAATGGTCACACAGC | CACTTGGCCGTTCTTTCACA |
| Os01g0618900 | TGTTGAAGGACTTGTGGGGT | CATGTGGAAGAAGGGCACAC |
| Os01g0620100 | CTTTGTGGGAGGAGGTCAGT | TTCCAACGAGTCCAAGAGCT |
| Os01g0621200 | CTTTCTCCCAATGGTCCCCT | CGCAGGGAATGGAATGATGG |
| Os01g0621300 | ATCGGCAGTATCAAGGGAGG | AGACAGATGGCGACCTTTCA |
