# Supplementary material for: Genome-Wide Association Mapping for Cold Tolerance in a Core Collection of Rice (Oryza sativa L.) Landraces by Using High-Density Single Nucleotide Polymorphism Markers From Specific-Locus Amplified Fragment Sequencing
Source: Front Plant Sci. 2018 Jun 28;9:875. doi: 10.3389/fpls.2018.00875 (PMC6036282; doi:10.3389/fpls.2018.00875)
Supplement: Supplementary file 2 [file Table_2.docx]

| No. | Chr | SNP number | Max P-alue | Min P-alue | Known loci | Gene function |
| --- | --- | --- | --- | --- | --- | --- |
| QTL 1 | 1 | 4 | 2.75E-07 | 1.66E-06 | - | - |
| QTL 2 | 1 | 3 | 9.88E-10 | 7.42E-06 | - | - |
| QTL 3 | 1 | 10 | 6.59E-07 | 5.89E-06 | - | - |
| QTL 4 | 2 | 4 | 2.03E-06 | 8.16E-06 | - | - |
| QTL 5 | 2 | 4 | 1.54E-06 | 3.84E-06 | OsFAD2 | Cold tolerance |
| QTL 6 | 2 | 10 | 1.80E-06 | 5.90E-06 | - | - |
| QTL 7 | 2 | 4 | 2.82E-06 | 5.76E-06 | - | - |
| QTL 8 | 2 | 7 | 1.77E-06 | 9.53E-06 | - | - |
| QTL 9 | 2 | 8 | 1.08E-06 | 6.44E-06 | - | - |
| QTL 10 | 2 | 6 | 1.08E-06 | 5.56E-06 | - | - |
| QTL 11 | 2 | 17 | 9.89E-07 | 1.06E-06 | - | - |
| QTL 12 | 2 | 6 | 1.10E-06 | 6.11E-06 | - | - |
| QTL 13 | 2 | 7 | 1.63E-06 | 5.22E-06 | - | - |
| QTL 14 | 3 | 7 | 1.43E-06 | 9.84E-06 | - | - |
| QTL 15 | 3 | 9 | 2.87E-07 | 1.14E-06 | - | - |
| QTL 16 | 3 | 11 | 1.38E-07 | 8.36E-06 | - | - |
| QTL 17 | 3 | 17 | 5.80E-07 | 7.12E-06 | OsMYB2 | Cold tolerance |
| QTL 18 | 3 | 9 | 2.75E-06 | 7.15E-06 | OsCIPK03 | Cold tolerance |
| QTL 19 | 3 | 5 | 6.63E-07 | 4.50E-06 | - | - |
| QTL 20 | 3 | 3 | 1.97E-06 | 5.20E-06 | - | - |
| QTL 21 | 9 | 3 | 1.25E-07 | 9.62E-06 | - | - |
| QTL 22 | 12 | 5 | 6.79E-07 | 6.59E-06 | - | - |

Table S2. 22 QTLs were mapped on 12 chromosomes.
