# Supplementary material for: Genome-Wide Association Mapping for Cold Tolerance in a Core Collection of Rice (Oryza sativa L.) Landraces by Using High-Density Single Nucleotide Polymorphism Markers From Specific-Locus Amplified Fragment Sequencing
Source: Front Plant Sci. 2018 Jun 28;9:875. doi: 10.3389/fpls.2018.00875 (PMC6036282; doi:10.3389/fpls.2018.00875)
Supplement: Supplementary file 3 [file Table_3.docx]

Table S3. Significant trait-marker associations with the Bonferroni threshold at p=0.01 (with an empirical p=1*10^-7^) for cold tolerance at seedling stage using the compressed mixed linear model (MLM) and 150 accessions of landraces in the Ting’s rice core collection.

| **Chromosome** | **SNP position** | **p-value** | **R2** |
| --- | --- | --- | --- |
| chr01 | 6362482 | 1.55E-09 | 0.28 |
| chr01 | 26297784 | 9.88E-10 | 0.27 |
| chr01 | 35276904 | 1.56E-08 | 0.26 |
| chr03 | 11082317 | 1.38E-07 | 0.32 |
| chr03 | 13410180 | 1.04E-09 | 0.30 |
| chr04 | 19624458 | 1.68E-09 | 0.28 |
| chr04 | 20440388 | 2.97E-09 | 0.27 |
| chr05 | 11508628 | 1.39E-09 | 0.28 |
| chr06 | 587507 | 1.18E-09 | 0.29 |
| chr06 | 1320300 | 1.25E-09 | 0.28 |
| chr06 | 11916477 | 1.57E-09 | 0.30 |
| chr06 | 20093648 | 1.71E-09 | 0.33 |
| chr07 | 322940 | 5.66E-10 | 0.29 |
| chr07 | 10449194 | 1.41E-09 | 0.32 |
| chr08 | 374729 | 6.26E-08 | 0.29 |
| chr08 | 2824184 | 1.41E-09 | 0.29 |
| chr08 | 23006565 | 1.12E-09 | 0.28 |
| chr08 | 27990046 | 7.57E-10 | 0.30 |
| chr09 | 6720639 | 2.38E-09 | 0.28 |
| chr09 | 13056377 | 1.25E-07 | 0.27 |
| chr10 | 3365663 | 1.06E-07 | 0.28 |
| chr10 | 4950360 | 1.08E-09 | 0.30 |
| chr10 | 5340485 | 1.52E-09 | 0.29 |
| chr10 | 5964213 | 1.27E-09 | 0.32 |
| chr11 | 5317840 | 1.60E-09 | 0.29 |

R2 indicates explained percentage by the QTL to the phenotypic variation using the compressed MLM.
